# Combined stomach and duodenal perforating injury following blunt abdominal trauma: a case report and literature review

**DOI:** 10.1186/s12893-020-00882-w

**Published:** 2020-10-02

**Authors:** Chun-Chi Lai, Hung-Chang Huang, Ray-Jade Chen

**Affiliations:** 1 Division of Acute Care Surgery and Traumatology, Department of Surgery, Taipei Medical University Hospital, Taipei City, Taiwan; 2 Division of General Surgery, Department of Surgery, Taipei Medical University Hospital, Taipei City, Taiwan

**Keywords:** Blunt abdominal trauma, Stomach perforation, Duodenum perforation, Handle-bar injury, Retroperitoneal hematoma

## Abstract

**Background:**

Gastrointestinal injury following blunt abdominal trauma is uncommon; a combined stomach and duodenal perforating injury is even more rare. Because these two organs are located in different spaces in the abdomen, such injuries are difficult to identify.

**Case presentation:**

A young woman involved in a motor vehicle crash presented to our emergency department with concerns of severe peritonitis. Contrast-enhanced computed tomography of the abdomen revealed pneumoperitoneum and retroperitoneal hematoma in zone 1. An emergency laparotomy was performed, revealing a stomach-perforating injury, which was resolved with primary repair. No obvious injury was observed on retroperitoneal exploration. However, peritonitis presented again on the second postoperative day, and a second laparotomy was performed, revealing a duodenum-perforating injury in its third portion. We performed primary repair with multi-tube-ostomy. The patient recovered well without permanent tube placement or internal bypass.

**Conclusions:**

Assessing associated injuries in blunt abdominal trauma is crucial because they may be fatal if timely intervention is not undertaken. These types of complicated injuries require a feasible surgical strategy formulated by experienced surgeons, which gives the patient a better chance of survival.

## Background

Traumatic gastrointestinal injury is an injury to the stomach, duodenum, small bowel, or colon following blunt or penetrating abdominal trauma. In patients who have sustained blunt abdominal trauma, the incidence rates of gastrointestinal, stomach, and duodenal injuries are 0.81–3.1%, 0.1, and 0.4%, respectively, according to previous studies [1–3]. Moreover, a perforating injury of both the stomach and duodenum following abdominal trauma has rarely been reported. High morbidity and mortality rates have been reported for patients with traumatic gastrointestinal injury and have been associated with misdiagnosis, severe intra-abdominal infection, and sepsis. In combined-organ injuries, we could easily be distracted by one injury and overlook the other. Therefore, accurate diagnosis and timely surgical intervention are crucial. We report a rare case of a combined stomach and duodenal perforating injury.

## Case presentation

**Fig. 1:**
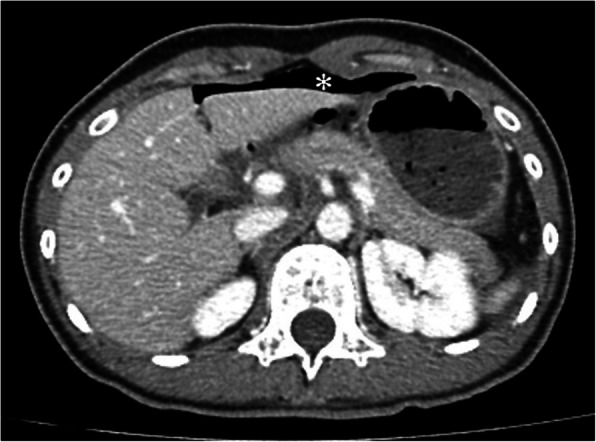
Massive extraluminal free air in the peritoneal cavity

**Fig. 2:**
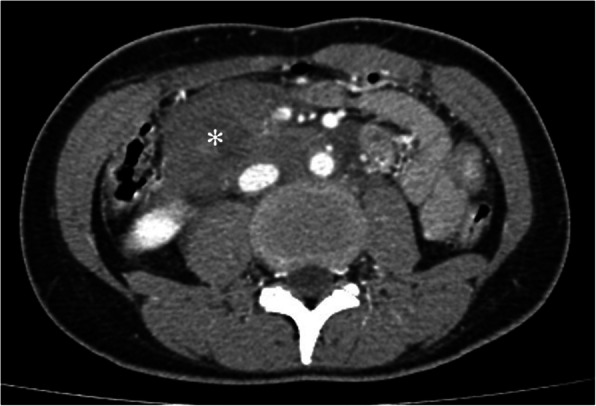
Zone 1 retroperitoneal hematoma

**Fig. 3:**
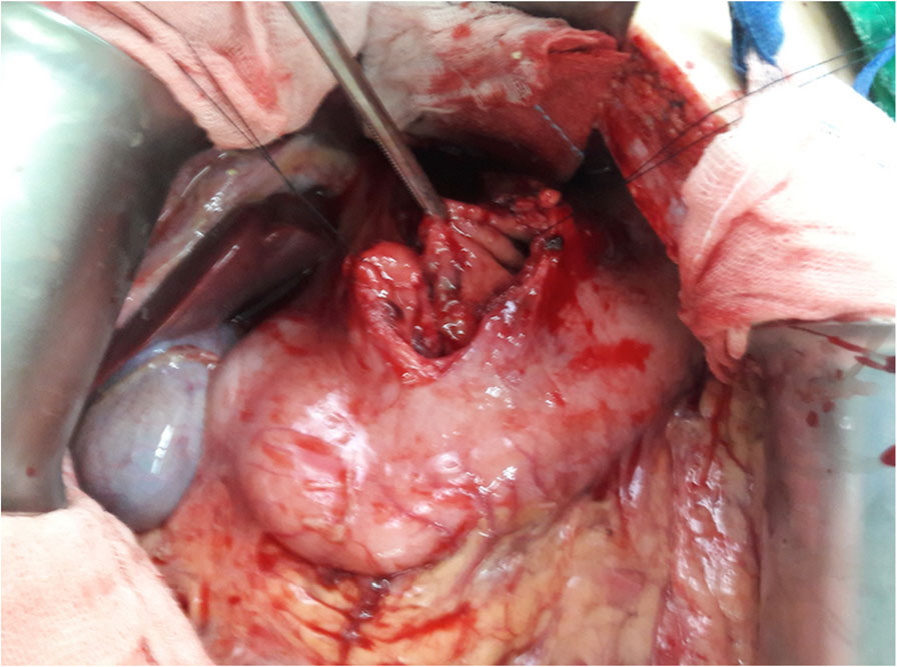
Grade III stomach injury of the lesser curvature (> 10 cm)

**Fig. 4:**
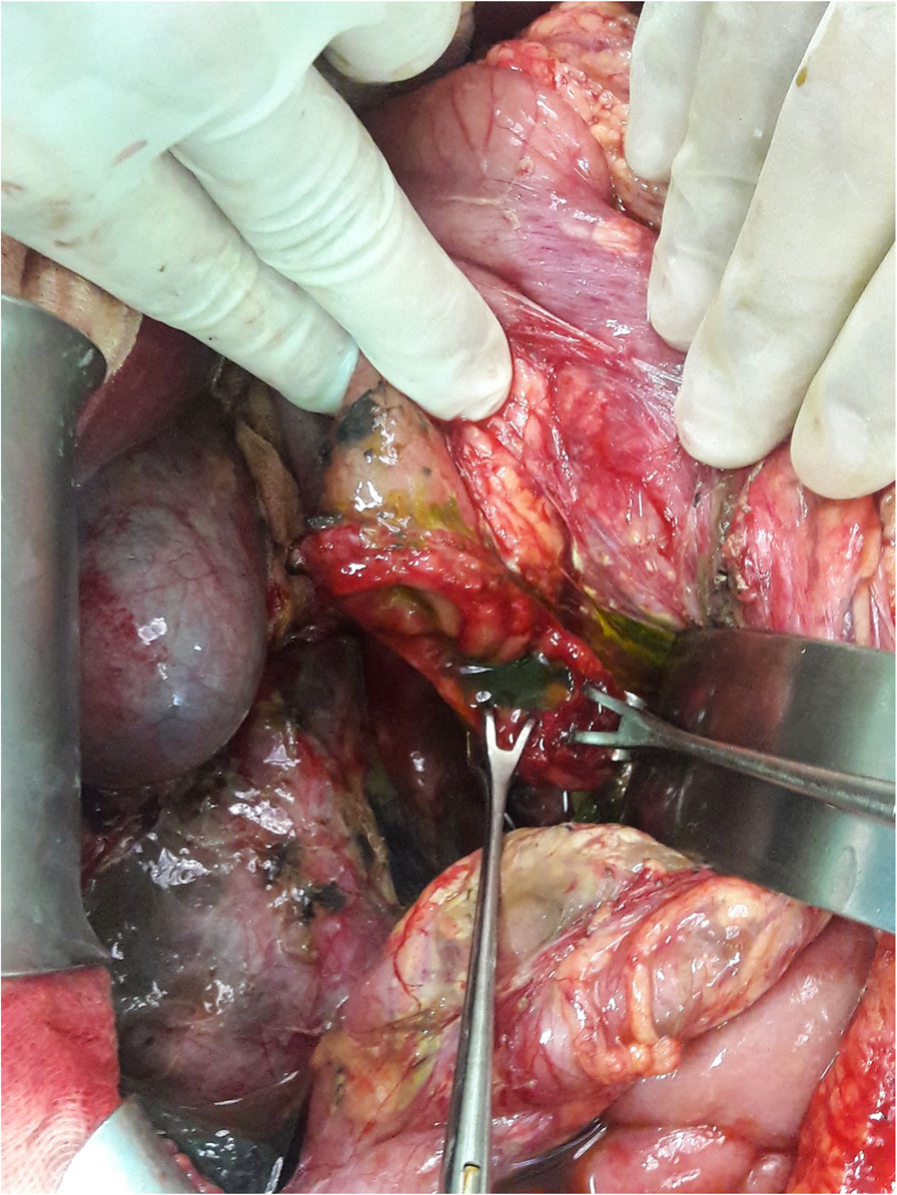
Grade III duodenal injury in its third portion (near complete transection)

**Fig. 5:**
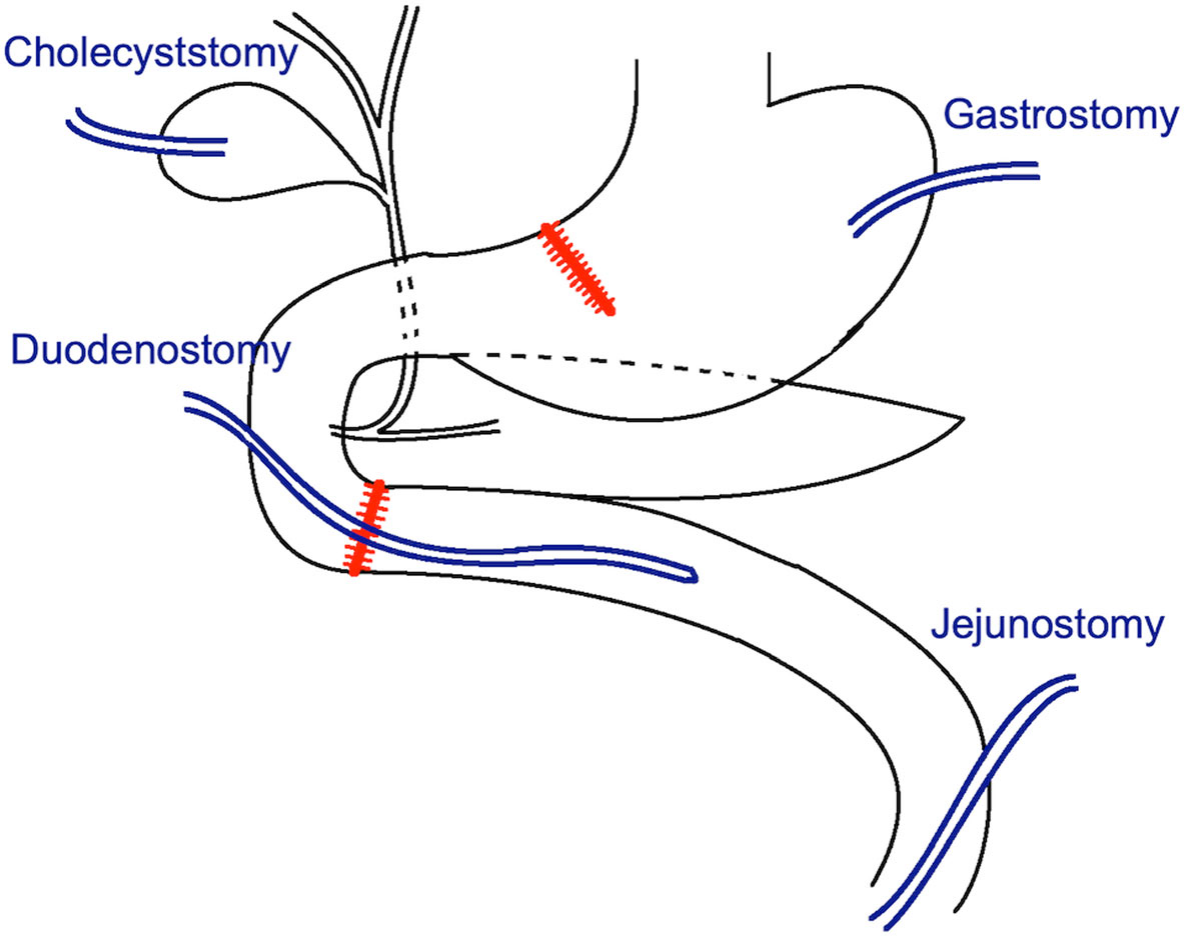
Surgical design (primary repair of stomach and duodenal injuries, digestive juice decompression, and enteral nutrition access)

**Fig. 6:**
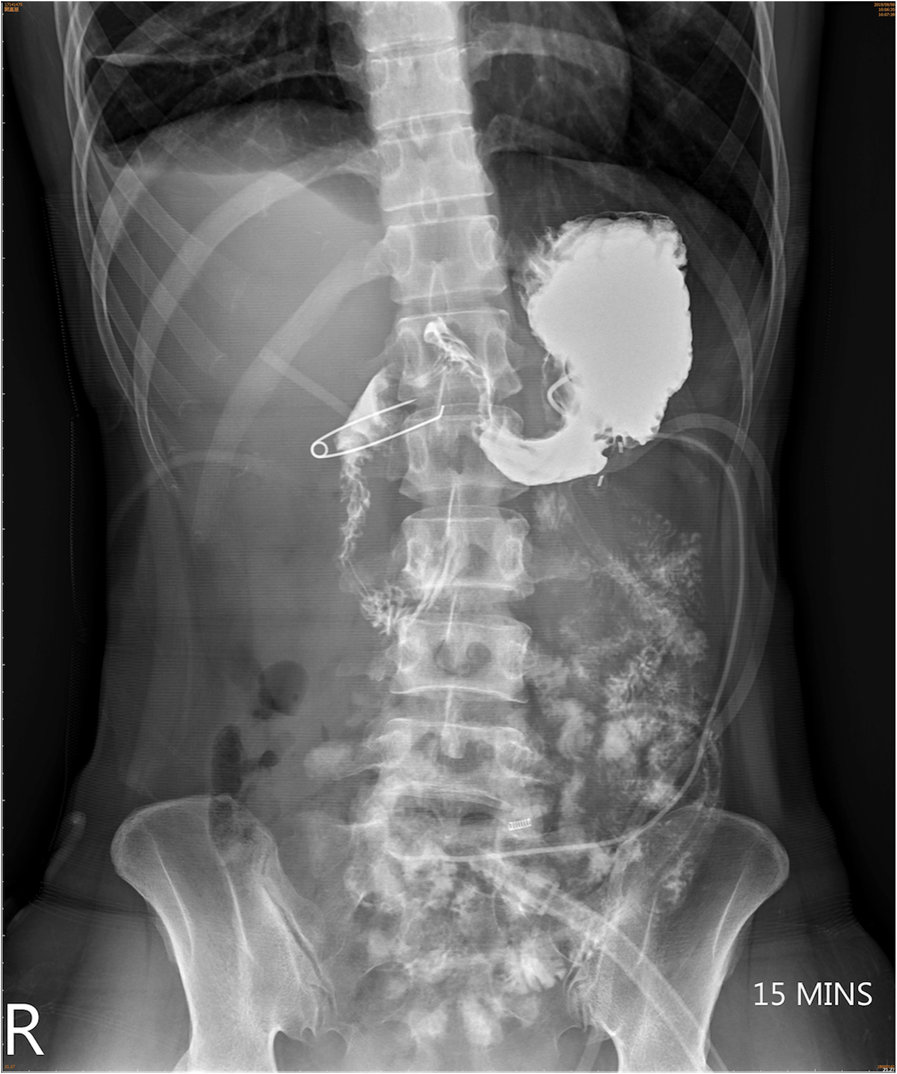
The continuity of the entire alimentary tact was unchanged without any permanent tube-ostomy or internal bypass after the patient recovered

A 33-year-old female motorcyclist presented to our emergency department with a 3-h history of severe abdominal pain following blunt abdominal trauma after bumping by a car. She denied any medical or surgical history. We evaluated this patient according to the advanced trauma life support algorithm. The airway was patent, and bilateral breath sounds were clear. The heart rate was mildly elevated to 100 bpm without low blood pressure. She was conscious and clear without any neurologic deficits. No obvious open wounds were observed, except for an erythematous bruise on the right middle abdomen, measuring 3 × 3 cm in size. Contrast-enhanced computed tomography (CT) revealed pneumoperitoneum with mild ascites and a retroperitoneal hematoma in zone 1 (Figs. 1 and 2). We performed an emergency exploratory laparotomy suspecting hollow organ perforation and retroperitoneal hematoma. On midline laparotomy, we noted that a considerable amount undigested food had spilled out of the distended stomach onto the gastrohepatic ligament and lesser sac. A transverse full-thickness laceration on the lesser curvature of the stomach was observed, measuring approximately 10 cm in length (Fig. 3). On retroperitoneal exploration, we used the Kocher maneuver to examine the first and second portions of the duodenum and observed them to be intact. We then opened the lesser sac to examine the pancreas and fourth portion of the duodenum, which were intact as well. No bilious ascites or active bleeding was noted during retroperitoneal exploration. Therefore, we performed a primary repair of the perforated stomach and sent the patient to the intensive care unit for further resuscitation. However, on the second postoperative day, peritonitis presented again, and the drainage tube showed bilious content. Hence, a second laparotomy was performed. This time, however, we observed bilious ascites in the lower abdomen. The repaired perforated stomach was intact. We then performed a complete right medial visceral rotation (Cattell–Braasch maneuver) and observed a nearly complete transection perforation on the third portion of the duodenum (Fig. 4). Hence, we performed a primary repair of the perforated duodenum. We also performed gastrostomy, duodenostomy, and cholecystostomy to relieve compression engendered by digestive juices. A jejunostomy procedure was also performed for enteral nutrition access (Fig. 5). However, duodenal leakage was noted on the ninth postoperative day. We controlled sepsis with resuscitation, antibiotics, and thorough drainage of intra-abdominal abscess. Total parenteral nutrition was also administered until sufficient enteral nutrition via the jejunostomy was achieved, which took approximately 1 week. Spontaneous closure of enterocutaneous fistula occurred 5 weeks later. She stabilized gradually, and drainage tubes were removed individually. She was discharged on the 90th postoperative day. All drainage tubes and all tube-ostomy bags were removed. The continuity of the alimentary tact was unchanged without any permanent tube-ostomy or internal bypass (Fig. 6). She showed complete recovery at another 3-month outpatient department follow-up.

## Discussion and conclusions

Gastrointestinal injury following blunt abdominal trauma is rare [1–3]. One mechanism underlying the onset of such injury involves the compression of a hollow viscus organ against the rigid part of human body (such as vertebra or thoracic cage) because of an external force (such as a force exerted by a seatbelt, handlebar, or steering wheel). Another mechanism involves shearing between the fixed and movable parts of the hollow viscus organ due to sudden deceleration during vehicle braking. According to the EAST (the Eastern Association for the Surgery of Trauma) Hollow Viscus Injury Study, the small bowel is the most commonly injured organ in hollow viscus organ injuries, followed by the colon, duodenum, stomach, and appendix [2]. Our patient presented with severe abdominal pain after sustaining blunt abdominal trauma caused by a motor vehicle crash. According to her statement, she had just finished eating when the crash occurred; hence, her stomach was distended, which is one of the risk factors for stomach injury following blunt abdominal trauma [4]. Moreover, the only wound was the erythematous bruise on the right middle abdomen, measuring 3 × 3 cm in size, which indicated a handlebar injury. A handlebar injury causing duodenal perforation is more common in children; only a few relevant cases have been reported in adults [5]. A CT scan provides accurate assessment for patients with trauma, such as the severity of injury in the peritoneal and retroperitoneal spaces. In our patient, CT revealed pneumoperitoneum and a retroperitoneal hematoma in zone 1. Pneumoperitoneum indicates hollow organ perforation in the peritoneal cavity and requires a laparotomy [6]. In laparotomy for traumatic injury, the first goal is to stop bleeding; the second goal is to identify gastrointestinal injury. Furthermore, a retroperitoneal hematoma indicates injury to the retroperitoneal organs or great vessels. The retroperitoneum is categorized into three zones. Zone 1 represents the central retroperitoneum, bordered by the aortic hiatus superiorly, sacral promontory inferiorly, and bilateral renal hila on the sides. Zone 1 contains the abdominal aorta, inferior vena cava, duodenum, and pancreas [7]. For a zone 1 retroperitoneal hematoma, an exploratory laparotomy becomes mandatory in both penetrating and blunt injuries because of the possibility of injury to the vasculature, duodenum, or pancreas. Zone 1 retroperitoneal exploration can be managed with the Kocher maneuver to examine the first and second portions of the duodenum. Subsequently, a right medial visceral rotation (Cattell–Braasch maneuver) can be performed to examine the inferior vena cava, infrarenal aorta, third portion of the duodenum, and head of the pancreas. The lesser sac could be opened to inspect the body and tail of the pancreas [8, 9]. For our patient, we had to check not only intraperitoneal but also retroperitoneal spaces. Approaching the third portion of the duodenum requires considerable effort and skill because it is hidden deep in the retroperitoneum and surrounded by vital structures. However, if no reasonable explanation for a zone 1 retroperitoneal hematoma is obtained after the first, second, and fourth portions of the duodenum and pancreas are examined, then exposing the third portion of the duodenum is essential. We ultimately identified a combined grade III (AAST) perforating injury of the stomach and duodenum during surgery [10, 11].

The type of surgery is planned according to the severity of injury. The stomach is a well-vascularized organ; therefore, primary repair with an air leak test is widely performed for grade I, II, and III (AAST) injuries of the stomach [12]. Gastrectomy with reconstruction should be considered for tissue loss or devascularization [13]. Regarding duodenal injury, primary repairs can be performed for grade I and II (AAST) duodenal injuries. For grade III (AAST) duodenal injuries, various repair approaches can be used. Primary repair in tension-free fashion is the top choice of repair approach. Duodeno-duodenostomy can also be used if tension-free primary repair is not possible. Roux-en-Y duodeno-jejunostomy will be applied if previous 2 approaches are not possible [14, 15]. For grade IV and V (AAST) duodenal injuries, damage control or staged Whipple’s surgery can be considered [16]. Ancillary procedures will be performed for the specific circumstances. Duodenal diversion will be considered for tenuous duodenal repair. Feeding jejunostomy is a good access to build up early enteral nutrition. Periduodenal drains are not always required, but should be placed for tenuous duodenal repair or grade III (AAST) duodenal injuries [14]. We performed primary repair for the stomach injury in our patient. Concerning the duodenal injury, tension-free primary duodenal repair is a favorable repair approach. In our case, the tension of the approximation of the duodenal perforation was tolerable, therefore, primary repair was performed for the perforation. Nevertheless, because of the infected intra-abdominal environment and high leakage rate, ancillary procedures was also required for this case. There are 3 types of duodenal diversions, which are Berne’s duodenal diverticulization, pyloric exclusion and tube duodenostomy [14]. Since the stomach perforation has been repaired in the first operation, Berne’s duodenal diverticulization and pyloric exclusion were not possible to be done. Hence, we choose tube-ostomy for duodenal diversion. Furthermore, feeding jejunostomy and periduodenal drains were also performed. Consequently, we performed a primary repair with multi-tube-ostomy.

Duodenal injuries have been reported to have high morbidity and mortality rates (27.1 and 5.3%–30%, respectively). Morbidity was reported to be mostly caused by intra-abdominal abscesses (15%), followed by duodenal fistulae (6%). The mortality rate varied according to the severity of organ injury (AAST): grade I (8.3%), grade II (18.7%), grade III (27.6%), grade IV (30.8%), and grade V (58.8%) [17]. Risk factors for duodenal repair site leakage were reported to include the severity of organ injury and a time interval from injury to repair exceeding 24 h, which also increase morbidity and mortality [18]. Weale et al. reported that the leakage rate for a grade III duodenal injury treated with primary repair is as high as 66% [19]. In our patient, duodenal repair site leakage was noted on the ninth postoperative day. In the acute phase of intra-abdominal abscess, the first step is to control sepsis with adequate drainage and antibiotics. In the chronic phase, an enterocutaneous fistula is another problem following duodenal leakage. An enterocutaneous fistula originating from the upper gastrointestinal tract (proximal to the duodenojejunal junction) would be associated with a higher chance of spontaneous closure (73.3%) than would that originating from the lower gastrointestinal tract (35.3%) [20]. Nearly 90% of the fistular tract closes spontaneously in the first month, with the remaining 10% closing in the second month [21]. Nutritional supplements work favorably for spontaneous closure, and enteral nutrition is superior to parenteral nutrition. For patients with a high risk of leakage, digestive juice decompression, distal enteral nutrition access creation, and adequate drainage tube placement are warranted. Our patient showed a high-output enterocutaneous fistula with a daily enteric secretion of approximately 500 mL. Total parenteral nutrition was administered in the acute phase. After the sepsis was controlled, we started ramp enteral feeding through a feeding jejunostomy technique and prescribed somatostatin to suppress the fistular output. Spontaneous closure occurred 5 weeks later.

Accurate diagnosis of a combined stomach and duodenal injury following blunt abdominal trauma is challenging. For such complicated gastrointestinal injuries, a feasible surgical strategy formulated by an experienced surgeon is crucial. From our experience, for treating a combined stomach and duodenal perforating injury following blunt abdominal trauma, we conclude that a primary repair for the stomach injury and primary repair with multi-tube-ostomy for the duodenal injury would be a feasible approach.

## Data Availability

Not applicable.

## Abbreviations

CT: computed tomography
AAST: American Association for the Surgery of Trauma
